# Motor Skills and Visual Deficits in Developmental Coordination Disorder: A Narrative Review

**DOI:** 10.3390/jcm11247447

**Published:** 2022-12-15

**Authors:** Elena Pinero-Pinto, Rita Pilar Romero-Galisteo, María Carmen Sánchez-González, Isabel Escobio-Prieto, Carlos Luque-Moreno, Rocío Palomo-Carrión

**Affiliations:** 1 Department of Physical Therapy. Faculty of Nursing, Physiotherapy and Podiatry, University of Seville, 41009 Seville, Spain; 2 Department of Physiotherapy, Faculty of Science Health, University of Málaga, 29016 Málaga, Spain; 3 Department of Physics of Condensed Matter, Optics Area, University of Seville, 41012 Seville, Spain; 4 Instituto de Biomedicina de Sevilla (IBIS), 41013 Seville, Spain; 5 Department of Nursing, Physiotherapy and Occupational Therapy, Faculty of Physiotherapy and Nursing, University of Castilla-La Mancha, 45071 Toledo, Spain

**Keywords:** developmental coordination disorder, visual deficits, motor skills, review, motor skills deficits, vision impairments, motor performance

## Abstract

Background: Developmental coordination disorder (DCD) is a developmental disorder in which numerous comorbidities seem to coexist, such as motor and visual impairment and some executive functions; Methods: A narrative review on motor and visual deficits in children with DCD was carried out; Results and Discussion: Fine and gross motor skills are affected in children with DCD. In addition, they seem to be related to visual deficits, such as difficulty in visual perception, sensory processing and visual memory. Limitations have also been found in accommodation. Interventions in children with DCD should be aimed at improving both aspects, since vision affects motor skills and vice versa; Conclusions: In children with DCD, who present a marked deficit in global shape processing, it causes an association between deficiencies in visual perception and motor skills.

## 1. Introduction

Developmental coordination disorder (DCD) is a heterogeneous condition occurring in nearly 6% of the general population [1]. It appears that performance deficits may be related to functional and structural problems in a distributed neural network that supports motor control and learning [2].

The onset of symptoms is determined at an early age [3]. The main motor deficits described in the DSM-V are voluntary gaze control during movement, dependent motor training/learning, cognitive/motor integration and atypical motor network functioning [3]. Motor control deficits in DCD depend on the nature of the task to be performed. Deficits are evident for dual tasks and tasks that require greater temporal or spatial precision, or a more complex planning that requires some adaptation/adjustment at the perceptual-motor level to maintain stability [2]. In addition to motor impairments, which may affect all motor skills or only some motor skills [4,5,6], the literature reports other frequent impairments, such as visual [1,7,8], cognitive [9,10,11] and reduced executive functions [1,12].

In order to acquire proper motor skills, adequate visual feedback is necessary [13]. Children with DCD present some difficulties in sensory processing and integration [14,15], especially in visual perception [16,17]. These deficits in visual perception, as well as other visual disturbances present in children with DCD, appear to affect motor skills [15,16,18].

Children with visual deficits and a condition that affects their neurodevelopment may require extensive and specialized help, although there is no evidence on the most effective strategies for visual improvement in children with DCD [19]. The objective of this narrative review is to characterize the state of the art on visual deficiencies in individuals with DCD and their influence on motor deficits.

## 2. Materials and Methods

This narrative review presents an overview of the currently available literature regarding the epidemiology of visual and motor deficits in DCD and the intervention on these deficits. The study was based on reviews, original articles, meta-analyses and intervention guides published in English. 

### 2.1. Information Sources

A literature search was performed from 1 August 2022 to 30 September 2022 using Web of Science, PubMed, Scopus, and Cinahl. The search was performed by two reviewers separately. The searches were carried out in the time period from 2002 to 2022. Any disagreements between the two reviewers were resolved by a third unblinded reviewer. Articles were screened by title and abstract, and subsequently, the full texts of the selected articles were examined. 

### 2.2. Search Strategy

The literature search used various combinations of the keywords “developmental coordination disorder” AND “motor skills” OR “motor disorder” OR “motor skills disorders” OR “Motor Performance” in combination with one or more of the following: “vision”, “visual”, “vergence”, “strabismus”, “eye movements”, “phoria”, “stereovision”, “stereoacuity”, “refractive errors”, “vision impairments” and “visual acuity”. Those studies relevant to visual disturbances, motor disturbances and both deficits in combination with each other were selected, with the aim of identifying the relationship between them. Articles that were irrelevant to the scope of this review were excluded. Additional literature was identified from the reference lists cited in the initially identified articles.

### 2.3. Eligibility Criteria

The selection criteria included publications that described the visual characteristics of children with DCD, as well as those that described motor deficits in the same population, and those that included visual and motor variables in their intervention in these children. They had to be available in full text and written in English. Articles were excluded if: (1) they did not report data on motor and/or visual deficits in DCD; (2) the patients included were adults; (3) the article was a letter, conference abstract or study protocol. After applying electronic filters, duplicates and unintelligible articles were removed by including them in Mendeley (Mendeley Software, London, UK), which is a bibliographic software used to acquire and organize all references. Manual selection of titles and abstracts was performed immediately by two different reviewers. The selected eligible articles underwent a full-text review by two independent investigators.

### 2.4. Data Collection Process

Data were extracted using a standardized form, which included the following information: (1) names of the authors and year of publication, (2) type of study, (3) variable analyzed (visual/motor/both/intervention), and (4) relevant data.

### 2.5. Quality Assessment of Narrative Review Articles

To carry out the evaluation of this narrative review, we used the SANRA tool, which is a brief scale for the quality assessment of narrative review articles. SANRA’s internal consistency and item-total correlation are sufficient, with satisfactory inter-rater reliability. This tool consists of 6 items: explanation of the review’s importance (item 1) and statement of the aims (item 2) of the review, description of the literature search (item 3), referencing (item 4), scientific reasoning (item 5), and presentation of relevant and appropriate endpoint data (item 6). Two reviewers external to this study administered the SANRA tool to determine the quality of the study.

## 3. Results 

### 3.1. Selection of Sources of Evidence

**Figure 1:**
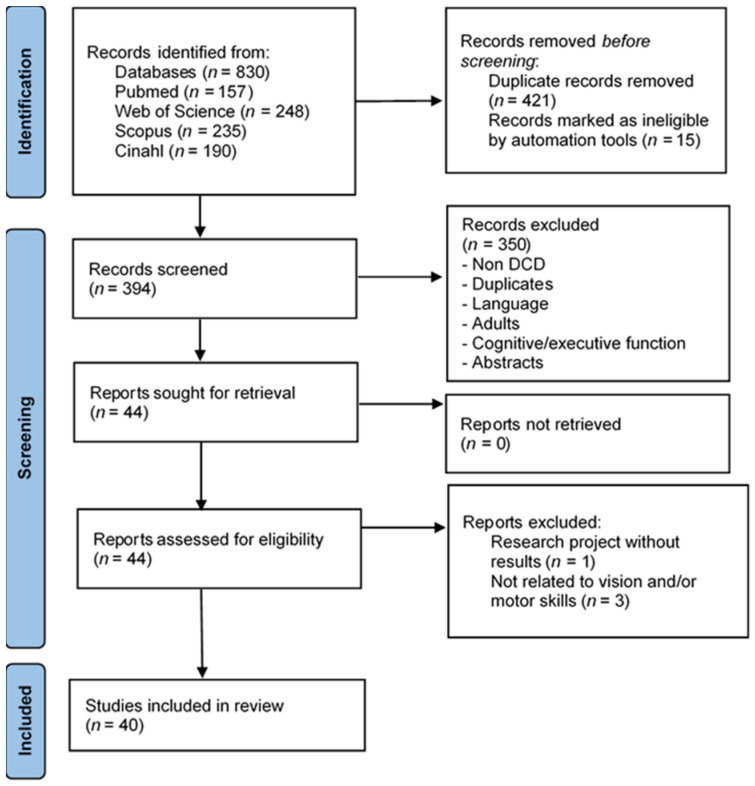
Schematic representation of the strategy for the selection of final articles.

The search in the database, using the keywords mentioned above without any filter, resulted in 830 documents. After removing duplicates and articles that could not be read by Mendeley, a total of 394 articles remained in the sample. The manual selection of titles and abstracts resulted in the exclusion of 350 studies, leading to 44 eligible studies. A total of 4 papers were excluded from the study, since the data they provided were not relevant in relation to visual and motor skills in children with DCD. The selected studies are shown in Figure 1. Most of the selected studies were descriptive and experimental studies.

### 3.2. Study Characteristics

A total of 7 articles addressed the motor skills of children with DCD with some involvement of the visual system [9,10,20,21,22,23,24]. Another 8 articles addressed the visual characteristics of children with DCD [25,26,27,28,29,30,31,32]. Of the 40 articles, 17 addressed the relationship between motor skills and vision in children with DCD, of which 10 investigated fine motor skills [4,8,33,34,35,36,37,38,39,40] and another 7 investigated gross motor skills [5,7,41,42,43,44,45]. The remaining 8 articles deal with visual intervention that influences the motor development of children with DCD [46,47,48,49,50,51,52,53]. Table 1 shows the specific characteristics of the selected studies, such as the type of study, the variable studied, and other relevant data. 

A total of 17 selected articles were descriptive studies, although some did not specify it [4,7,8,9,10,21,23,27,28,29,30,32,33,34,35,37,38,44]. Another 18 were experimental, including the RTCs [5,22,26,31,39,40,41,42,43,45,46,47,48,49,50,51,52,53]. The rest were other types of studies [20,24,25,36].

### 3.3. Quality Assessment of This Narrative Review Article

Two reviewers external to the research (MPS and JMSG) administered the SANRA tool to this narrative review to determine its quality level. The minimum score in each item is 0 and the maximum is 2, from lowest to highest quality in each item. The sum score of both reviewers is 11, with some difference that can be observed in Table 2.

## 4. Discussion

### 4.1. DCD Motor Skills 

Seven studies examine the motor characteristics of children with DCD [9,10,20,21,22,23,24]. Children with DCD have fine and/or gross motor skills below the level expected for their age and learning opportunities [9]. According to the diagnostic criteria of the DSM-V [3], people with DCD acquire motor coordination below expectations for their chronological age and present clumsiness, inaccuracy in the performance of motor skills or slowness. The motor deficit described seems to interfere with activities of daily living, academic activities, or age-related leisure activities, although it has not been related to a medical condition or disease. 

Research shows that children with DCD exhibit slower, more variable reaction times compared to typically developing children as a result of either slower processing speed, inefficient preparation of movement or both [15]. Motor planning appears to be impaired in DCD on most but not all tasks. Tests of visuomotor adaptation have shown that children with DCD present a lower capacity to adapt their movement to different task constraints. This has been shown by a higher movement variability, lower movement accuracy and/or longer movement durations [15]. Movement times or durations are frequently reported to be longer in children with DCD than in typically developing children, probably as a result of a stronger reliance on visual information for movement control [20]. 

Children with DCD also show delayed postural adjustment time [20]. Acquiring postural control requires the ability to integrate inputs from the somatosensory, visual, and vestibular systems and to use the integrated sensory signals to generate coordinated motor actions [42]. Opitz et al. [10] reported that children with DCD improved their reaction times when they were learning motor sequences, but showed less accuracy in distinguishing between different sequences. This impaired explicit discrimination was observed in different domains, including the visuospatial and temporal domain.

Motor control strategies to regulate muscle activity are less uniform and consistent than in typically developing children [42]. Different studies analyze postural patterns in children with DCD [5,21,22,42,50]. Alterations are found in the timing and pattern of activation of the postural muscles used to maintain posture during goal-directed reaching. The normal sequence of muscle activation from distal to proximal in disturbed standing was replaced by a pattern of activation from proximal to distal. Balance problems have also been found, with greater coactivation of leg muscles when standing on their non-preferred leg [21]. All of these neuromuscular deficits can affect the motor strategies used by these children for postural control.

Imitation and visual learning are essential for motor development; therefore, it is possible that imitation difficulties have an impact on the acquisition of movement in children with DCD [23]. To achieve a correct imitation, the integration of multiple sensory systems is required. That is why deficits in imitation could also be a consequence of dysfunction of processes that have also been associated with DCD, such as visual attention or processing, memory and executive function, sensory-perception function, or motor learning and adaptation. Furthermore, they are unlikely to be limited to a single area. 

The difficulty in acquiring movement skills in children with DCD may be due, among other things, to a deficit in imitation and observational learning. Motor control is the ability to initiate and produce intentional, coordinated and precise movements [24], which are aspects that can be affected by coordination deficits and imitation deficits.

There are few studies looking at limb function in children with DCD [36,37,38]. Deficits found in manual skills in children with DCD include slower reaction time, reduced accuracy, and more variable speed of movement when reaching [4,24,36]. In relation to bimanual coordination in children with DCD, the scientific literature suggests that the deficits may be more evident in the non-dominant limb, in addition to the fact that children with DCD may also show difficulty with coupling between limbs [24]. In the most difficult bimanual tasks, bilateral deficits in spatiotemporal metrics are observed in children with DCD [46].

### 4.2. DCD Visual Deficits

A total of 8 studies have analyzed the visual disturbances that occur in children with DCD [25,26,27,28,29,30,31,32]. Children with severe DCD have abnormalities in binocular vision, refractive errors, and ocular alignment [25]. Accommodation abnormalities, which contribute to impaired motor skills in children with DCD, have also been found [33,34]. The study developed by Bilbao and Piñero [26], resolves that children with DCD have a significantly lower amplitude of accommodation and a trend of greater exophoria compared to those with other developmental disorders.

Sumner et al. [27] found deficits in maintaining participation in fixation and following tasks with more antisaccade errors in a group of children with DCD compared to a control group. Some studies [25,28] that analyzed eye-tracking records showed abnormal eye movements in children with DCD (on screen, the number of fixations was higher and the duration of each fixation was shorter in children with DCD than in control children). These children also made more saccadic eye movements. However, other authors [30] found no relationship between the imprecision of eye movements and the imprecision of numerical estimation in children with DCD. Gonzalez et al. [29] explain how cognitive control influences saccadic eye movements in children with DCD. It appears that these children are competent in executing saccades during reflexive conditions (without cues), but show deficiencies in more complex control processes involving prediction and inhibition.

In typically developing children, soft horizontal seeking is mature by the age of 7 years, whereas soft vertical seeking is not mature until late adolescence. Robert et al. [28] hypothesize that children with DCD have a late maturation of both search systems. In their study, horizontal pursuit gain was similar in both populations, but vertical pursuit gain was significantly impaired, that is, it was more saccadic in children with DCD than in typically developing children. Some atypical ocular motility has been identified in patients with DCD [27], especially with regard to poor sustained engagement in fixation in DCD subjects. There also appear to be differences in gaze behavior compared to control groups [37]. Gaze training was investigated to verify whether it retrospectively generated benefits in movement organization [53].

Children with DCD often have deficits in sensory processing and visual perception [9,14,18,31,50,54]. Children with DCD perform significantly worse on the visual perception test compared to typically developing children, although the deficits are not common to all children with DCD. This means that there is great variability in the visuo-perceptive clinic of children with DCD [17]. Nevertheless, Crawford and Dewey [32] suggest that DCD alone is not associated with visual perception problems. According to these authors, the presence of concurrent disorders could be the key to visual perception deficits in children with DCD. However, the number of concurrent deficits present in DCD is associated with the severity of visual perception dysfunction. For example, deficits in visual memory skills appear to be a specific area of difficulty for children with DCD and concurrent reading disability and/or attention deficit hyperactivity disorder.

### 4.3. Relationship between Vision and Motor Skills

Of the selected studies, we found 17 that relate vision and motor skills, 10 of them in relation to fine motor skills [4,8,33,34,35,36,37,38,39,40] and the other 7 on gross motor skills [5,7,41,42,43,44,45]. In order to acquire proper motor skills, adequate visual feedback is necessary [13]. It is quite possible that impaired visuomotor integration could be a phenomenon that affects the results in all tasks. The most frequently observed deficit in DCD involves the processing of visual information, which is an aspect that determines motor behavior [20]. Children with DCD use somatosensory information for postural control as effectively as children with normal development. Somatosensory function normally matures at the age of 3–4 years and is not affected by DCD, as the results of Fong et al. [42] demonstrate. Thus, children with DCD partially compensate their balance problem by relying on somatosensory input. Visual-spatial processing and visual-kinesthetic integration are prerequisites for the successful maintenance of stability, and they are usually impaired in children with DCD [5,42,45]. 

In this line, the study carried out by Cheng et al. [6] examined the extent to which the motor deficits of children with DCD, evaluated with the Movement Assessment Battery for Children-2 (MABC-2), are linked to their visual perception abilities. Results indicated that poor performance within DCD on tasks such as static visual discrimination, visual sequential memory and eye-hand sequential coupling will negatively affect performance in MABC-2 or in tasks of daily living. For typically developing children, visual perceptual skills did not correlate with their motor skills. Based on these results, children with DCD may have trouble coordinating visual cues to perform motor tasks. A similar study was carried out by Van Waelvelde et al. [16]. In this case, children with DCD also performed significantly worse than the control group on all measures. The visual discrimination task was not significantly correlated with any of the motor tasks. In this case, the association between visual perceptual deficits and motor tasks was shown to be task-specific. 

On the other hand, the relationship between accommodation and motor tasks in children with DCD has also been studied [33,34]. These children had significantly poorer accommodation facility and amplitude dynamics compared to the control group. Therefore, the results indicate a relationship between the alteration of accommodation and motor skills; more specifically, accommodation abnormalities were correlated with the performance of visuomotor, upper extremity and fine dexterity tasks [33]. Children with DCD exhibit reliance on accommodative feedback only on visuomotor and upper extremity tasks. Thus, children with DCD may be less dependent on visual feedback obtained from accommodation, as they have adaptive mechanisms to overcome faulty information when there are oculomotor abnormalities [34].

Micheletti et al. [54] suggest that two distinct visually related components, associated with global shape and global motion sensitivity, contribute to DCD differently across the range of severity of the disorder. In their study, the results within the DCD group indicate that the relationship between motor skill deficits and global visual perception is more complex than the comparison with typically developing controls indicates. When a marked deficit in global shape processing is present, as occurs in children with DCD, this dominates the association between deficits in visual perception and motor skills.

#### 4.3.1. Vision Deficits and Fine Motor Skills

A total of 10 articles were selected based on the theme of fine motor skills and vision [4,8,33,34,35,36,37,38,39,40]. The scientific literature shows that manual functions are more affected by vision and its effects in children with DCD [34]. Manual motor tasks, with visual support, which requires predictive control, are affected in children with DCD [18]. Catching performance in children with DCD probably reflects a combination of errors in paying attention to visual information and organization of movement [35]. Children with DCD are less able to use a predictive strategy during visuo-manual follow-up with intermittent occlusion. They are also less proficient at tracking a moving target than typically developing children. Ferguson et al. [18] showed that children in the DCD group made more changes to their trajectory and more recovery movements. Moreover, tracking performance deteriorates when visual feedback is reduced. Other authors [36] also defend that certain deficits, such as DCD, present abnormal visuomanual actions, which are observed in bimanual coordination and in the visual guidance of the action in the task and failures in motor planning. Initially, the study of Arthur et al. [37] does not agree with this fact, since they did not find evidence to support the proposition that children with DCD coordinate their hands and eyes in a non-predictive way. However, in a later exploratory follow-up analysis, they did find differences in fundamental eye movement patterns between groups, and children in the DCD group showed some evidence of atypical visual sampling strategies and gaze-anchoring behaviors during the task.

Regarding eye-hand coordination in children with DCD, the study by Wilmut et al. [38] concludes that there is no evidence of a problem in the speed or precision of simple movements, although they observed difficulty in linking sequential changes of gaze and hand required to complete everyday tasks or typical assessment items. Along the same lines, Grohs et al. [24] reported that children with DCD presented greater variability in the speed of the dominant limb, as well as greater deviations from the ideal trajectory of the non-dominant limb. Similarly, when reaching for a target, the trajectories followed by the DCD group were longer and more curved than those of the control group in the study of Zoia et al. [4]. Moreover, deceleration times were longer for the DCD group. This study concludes that the use of visual feedback by children with DCD may be different from that of typically developing children. However, it seems that there is no consensus on the relationship between the deficit in visual perception and handwriting skills. Prunty et al. [8] examined the role of visual perception and visuomotor integration in the identification and explanation of writing difficulties (speed, legibility, and excessive pauses) in children with DCD; these authors found that, although the DCD group scored poorly on measures of visual perception, these were not predictive of their handwriting performance. 

Overall, increased visual bias has been found to correlate with poor manual dexterity in children with DCD [40]. The study of Nobusako et al. [39] demonstrated that DCD children with clumsy manual dexterity have deficits in visuomotor temporal integration and automatic imitation function. In addition, they revealed a significant correlation between manual dexterity and visuomotor temporal integration measures. The results indicated that visuomotor temporal integration is the strongest predictor of poor manual dexterity.

#### 4.3.2. Vision Deficits and Gross Motor Skills 

Seven articles were found that related gross motor skills and vision [5,7,41,42,43,44,45]. According to Bair et al. [41], the postural body schema and the development of the dorsal stream are useful in explaining the reweighting of low vision. The lack of multisensory fusion supports the notion that optimal multisensory integration is a slow developmental process and is vulnerable in children with DCD. 

Among the gross motor skills, balance or stability in standing posture stands out. Several studies have explored the influence of vision on standing balance in children with DCD [5,21,22,41,42,43]. For example, Cherng et al. [5] showed that the standing stability of children with DCD was significantly poorer than that of control children subjected to different sensory conditions (visual and somatosensory inputs). The results suggest that children with DCD experience more difficulty in coping with altered sensory input, which has also been reported by Deconinck et al. [45]. In all conditions that the children were subjected to, the mean postural sway velocity was greater for children with DCD. It also revealed a greater reliance on vision in children with DCD when standing on a firm surface. These results suggest that postural control problems may still be associated with difficulties reappraising sensory information in response to environmental demands. Tsai et al. [21] were more precise in their study, since they discriminated between the dominant and non-dominant leg and between sexes. DCD children showed more difficulty standing on the non-dominant leg with their eyes open and closed. In addition, while the boys showed results similar to those of the total group, the girls with DCD only obtained significant differences in three conditions with eyes closed, but not with eyes open. Geuze [22] corroborates these results. In their study, DCD children had more difficulty standing on one leg with their eyes closed. While standing on the non-preferred leg, the DCD children’s electromyograms showed slightly greater coactivation of lower and upper leg muscles. If standing was disturbed, children with DCD took longer to regain their posture. However, children with DCD learned to compensate for the disturbance in a few attempts. In difficult or novel situations, children with DCD appear to suffer from increased postural sway as a result of suboptimal balance control. To facilitate standing postural control, Bair et al. [43] suggest that children with DCD benefit from the use of vision in combination with tactile information, possibly due to their less developed internal models of body orientation and self-motion. Internal model deficits, among other postural deficits, may increase balance impairment in children with DCD.

According to Tsai [7], DCD children performed significantly worse than the control group, although only visual perception and motor skills with timed responses were significantly correlated. Therefore, visual perception related to motor performance has a speed component in these children. This is also related to the results of Deconinck et al. [44] regarding the relationship of vision and gait in children with DCD. These authors suggest that children with DCD are more dependent on global visual flow information than typically developing children for maintenance of balance and speed control during gait. This increased reliance on visual control could be associated with an underdeveloped internal sensorimotor model.

### 4.4. Interventions for Vision and Motor Deficits in DCD

Of the selected studies, 8 refer to visual intervention as a therapy that directly influences the motor skills of children with DCD [46,47,48,50,51,52,53]. Children with DCD who meet diagnostic criteria generally need treatment. The indications for intervention essentially depend on the influence of the diagnosis on activities of daily living. However, in some cases, the diagnosis does not indicate treatment [2].

If DCD requirements are met but there are motor problems in performing tasks of daily living and in educational and social support, then strategies for participation in all environmental contexts should be implemented. This is common in children under 5 years of age who have significant motor problems but do not meet all the diagnostic criteria for DCD. Recommendation 17 of the International clinical practice recommendations on intervention in children with DCD is that evidence of effectiveness, including regimen and dose, should be considered when planning intervention. In the case of co-occurring disorders, they recommend that intervention priorities be established according to the type and severity of each disorder, and in consultation with the child and family [2].

There are different studies that analyze the effect of visual training to improve motor skills in children with DCD [46,47,48]. Quiet-Eye Training (QET) has been shown to be more effective than traditional training methods in teaching a throw and catch task. In the study of Miles et al. [47], QET improved DCD children’s ability to focus on a target on the wall before throwing, as well as better anticipation and tracking of the ball, which translates into better catching technique. QET could be an effective adjunct for therapists teaching visual-motor skills to children with DCD. Along the same lines, Norouzi Seyed Hosseini et al. [46] analyzed the effect of TQT on the bimanual coordination of children with DCD. The results indicated that the coordination mode performance was strongly influenced by the QET. Therefore, they conclude that the successful performance of a bimanual linear task depends mainly on the availability of visual feedback. Similarly, Wood et al. [48] have been shown to improve the ability to throw and catch a ball in children with DCD through TCE, which in turn also alleviates the negative psychosocial impact of these motor skill deficits. All parents of children with DCD in this study reported improvements in their children’s confidence, social skills, and predilection for physical activity after testing.

Coetzee and Pienaar [49] carried out a vision therapy program in children with DCD to verify whether visual motor problems improved. The vision therapy program lasted 18 weeks and was carried out once a week, with 40 min per session. A 75–100% improvement in visual tracking, fixation, ocular alignment, and convergence was reported in children with DCD, who underwent an 18-week vision therapy program [49].

The study of Nobusako et al. [39] suggested that improving visuomotor ability temporal integration can be an effective rehabilitation strategy for DCD. Therefore, it is necessary to develop new neurorehabilitation techniques that favor visuomotor temporal integration. In the same line, Fong et al. [50] showed that task-specific balance training marginally improves somatosensory function and somewhat improves balance performance in children with DCD. It appears that, by improving motor function, it is also possible to improve deficits in visual function.

In another sense, results suggest that motor imagery is accessible to children with DCD, but less refined/developed compared with healthy controls [20,51]. Different studies found that children with DCD were able to perform motor imagery, although with a slower and less accurate rate of mental transformation than controls [20,52,55,56]. The motor imaging deficit observed in children with DCD is associated with motor imaging accuracy, rather than vividness [56]. Generally, it seems that children with DCD may incorporate motor imagery adequately for simple tasks, but may use it less consistently than typically developing children. In motor planning tasks, it is important to systematically vary the complexity of motor imagery tasks to identify the specific capacities of the child with DCD [20]. As such, motor imagery can be voluntarily incorporated to reinforce the relationship between the (simulated) motor output signal and the resulting behavior of the physical system. The results of motor imagery training in improved skill and function are pervasive in motor learning. In a motor imagery training study, Wilson et al. [51] showed training effects comparable to conventional physical therapy. The mechanism of change could be related to the training of predictive models of action with repeated mental simulation.

### 4.5. Limitations and Strengths

Despite its narrative nature, the present study provides a comprehensive and systematic overview on visual disturbances in children with DCD and their possible influence on motor deficits.

However, we are aware that some documents may have been lost. A lack of consistent data in the reports, which are not always supported by numbers and statistics, suggests the need for more transparent and objective studies based on standardized reports. However, to our knowledge, this is the first study to describe related visual and motor impairments in children with DCD.

## 5. Conclusions and Clinical Implications

In children with DCD, the literature seems to indicate that there is a clear association between visual deficits and motor skills, both fine and gross. Future research should delve into this last relationship at the functional level to enable effective interventions. Due to the lack of homogeneity in the current studies on the relationship between vision and motor skills in children with DCD, more randomized clinical trials, as well as descriptive studies that analyze this relationship in depth, are necessary to establish a correct intervention for these children. It would be interesting for future research to use a fuller overview of methods, intervention and findings so that comparisons can be made between different studies.

## Figures and Tables

**Table 1:** Characteristics of the selected studies.

Author (Date)	Design	*n* (Mean Age, Years)	Outcome				
			Motor Skills	Visual Deficit	Relationship of Visual Deficit with		Visual/Motor Intervention
					Fine Motor Skills	Gross Motor Skills	
Van Dyck et al. (2022) [9]	D	50 (9)	•				
Adams et al. (2014) [20]	SR	-	•				
Opitz et al. (2020) [10]	D	24 (8.5)	•				
Tsai et al. (2008) [21]	D	64 (9.5)	•				
Geuze (2003) [22]	RCT	24 (9)	•				
Reynolds et al. (2017) [23]	D	29 (9.5)	•				
Grohs et al. (2021) [24]	CH	26 (10.6)	•				
Creavin et al. (2014) [25]	CS	7154 (7.5)		•			
Bilbao and Piñero (2021) [26]	ES	7 (9.5)		•			
Sumner et al. (2018) [27]	D	23 (8.9)		•			
Robert et al. (2014) [28]	D	27 (9)		•			
Gómez et al. (2017) [30]	D	20 (8.5)		•			
González et al. (2016) [29]	D	10 (10)		•			
Kagerer et al. (2004) [31]	ES	7 (8)		•			
Crawford and Dewey (2008) [32]	D	27 (8.8)		•			
Rafique and Northway (2015) [33]	D	9 (10.3)			•		
Rafique and Northway (2021) [34]	D	24 (10.4)			•	•	
Licari et al. (2018) [35]	D	11 (9.4)			•		
Braddick and Atkinson (2013) [36]	NR	-			•		
Arthur et al. (2021) [37]	D	19 (10)			•		
Wilmut et al. (2006) [38]	D	7 (7.5)			•		
Zoia et al. (2005) [4]	D	19 (9)			•		
Prunty et al. (2016) [8]	D	28 (11)			•		
Nobusako et al. (2018) [39]	ES	29 (9.8)			•		
Nobusako et al. (2021) [40]	ES	19 (9.3)			•		
Bair et al. (2012) [41]	ES	20 (9.1)				•	
Cherng et al. (2007) [5]	ES	20 (5)				•	
Fong et al. (2012) [42]	ES	22 (7.6)				•	
Bair et al. (2011) [43]	ES	11 (7.2)				•	
Deconinck et al. (2006) [44]	D	12 (7.8)				•	
Deconinck et al. (2008) [45]	ES	10 (7)				•	
Tsai et al. (2008) [21]	D	60 (10)				•	
Norouzi et al. (2021) [46]	ES	20 (8.5)					•
Miles et al. (2015) [47]	RCT	30 (9)					•
Wood et al. (2017) [48]	RCT	21 (8.6)					•
Coetzee and Pienaar (2013) [49]	CO	32 (7.9)					•
Fong et al. (2016) [50]	RCT	88 (7.7)					•
Wilson et al. (2016) [51]	RCT	54 (8)					•
Deconinck et al. (2009) [52]	ES	13 (9)					•
Slowinski et al. (2019) [53]	RTC *	21 (8.5)					•

*n*: sample size of children with DCD; D: Descriptive (not specified); SR: Systematic Review; RCT: Randomized Clinical Trial; CH: Cohort Study; CS: Cross sectional Study; ES: Experimental Study; NR: Narrative Review; CO: Cross-Over study; *: Pseudo-Randomized.

**Table 2:** Scores obtained by the two reviewers in the SANRA narrative review quality tool.

Reviewer	Item 1	Item 2	Item 3	Item 4	Item 5	Item 6	Sum Score
1	2	1	2	2	2	2	11
2	2	2	2	2	2	1	11

## Data Availability

Not applicable.
